# Zika Virus in Salivary Glands of Five Different Species of Wild-Caught Mosquitoes from Mexico

**DOI:** 10.1038/s41598-017-18682-3

**Published:** 2018-01-16

**Authors:** Darwin Elizondo-Quiroga, Aarón Medina-Sánchez, Jorge M. Sánchez-González, Kristen Allison Eckert, Erendira Villalobos-Sánchez, Antonio Rigoberto Navarro-Zúñiga, Gustavo Sánchez-Tejeda, Fabián Correa-Morales, Cassandra González-Acosta, Carlos F. Arias, Susana López, Rosa María del Ángel, Victoria Pando-Robles, Armando E. Elizondo-Quiroga

**Affiliations:** 1 Unidad de Biotecnología Médica y Farmacéutica, Centro de Investigación y Asistencia en Tecnología y Diseño del Estado de Jalisco, Guadalajara, Jalisco Mexico; 2 Secretaria de Salud Jalisco, Guadalajara, Jalisco Mexico; 3 Independent consultant in Ajijic, Jalisco, Mexico; 4 Dirección del Programa de Enfermedades Transmitidas por Vector, Centro Nacional de Programas Preventivos y Control de Enfermedades, Secretaría de Salud de México, Mexico City, Mexico; 5 Departamento de Genética del Desarrollo y Fisiología Molecular, Instituto de Biotecnología, Universidad Nacional Autónoma de México, Cuernavaca, Morelos, Mexico; 6 Departamento de Infectómica y Patogénesis Molecular, Centro de Investigación y de Estudios Avanzados del IPN, México City, Mexico; 7 Instituto Nacional de Salud Pública, Centro de Investigación Sobre Enfermedades Infecciosas (CISEI), Cuernavaca, Morelos, Mexico

## Abstract

Zika virus (ZIKV) is a mosquito-borne pathogen, and *Aedes aegypti* has been identified as the main vector of the disease. Other mosquito species in the *Aedes* and *Culex* genera have been suggested to have the potential for being competent vectors based on experimental exposition of mosquitoes to an infectious blood meal containing ZIKV. Here, we report the isolation in cell culture of ZIKV obtained from different body parts of wild-caught female mosquitoes (*Ae*. *aegypti*, *Ae*. *vexans*, *Cx*. *quinquefasciatus*, *Cx*. *coronator*, and *Cx*. *tarsalis*) and whole male mosquitoes (*Ae*. *aegypti* and *Cx*. *quinquefasciatus*) in Mexico. Importantly, this is the first report that shows the presence of the virus in the salivary glands of the wild-caught female mosquitoes species, *Cx*. *coronator*, *Cx*. *tarsalis*, and *Ae*. *vexans*. Our findings strongly suggest that all the species reported herein are potential vectors for ZIKV.

## Introduction

In 2015, Brazil was the first country in the Western Hemisphere to report Zika virus (ZIKV)^1^, but currently the transmission has spread to more than 50 countries and territories in the region^2^. As of September 2017, there were 9,987 confirmed cases of ZIKV infection, including 5,925 pregnant women, in 26 out of 32 states of Mexico^3^. ZIKV is a member of the *Flaviviridae* family and the genus *Flavivirus*; the presumptive primary vector of the virus is *Aedes aegypti* (L.), and laboratory studies have demonstrated its ability to acquire and potentially transmit the virus in mosquitoes experimentally fed with infected blood^4,5^. On the other hand, different laboratory studies have reported that although *Ae*. *aegypti* and *Ae*. *albopictus* (Skuse) are susceptible to acquire the infection, their level of competence and efficiency to transmit ZIKV ranges from 0 to 70 percent^6–9^. Furthermore, it has been recently shown that other mosquito species may also transmit the virus in laboratory conditions, including *Ae*. *vexans* (Meigen)^10,11^, and mosquitoes in the *Culex* genus, such as *Cx*. *quiquefasciatus* Say^12,13^.

To gain insight into the vector competence of different species in the metropolitan area of Guadalajara in the State of Jalisco, Mexico, we collected mosquitoes inside houses in neighborhoods where at least one confirmed or probable case of ZIKV in humans had been reported by the local health authorities. The ZIKV present in different body parts of the mosquitoes was propagated by cell culture and the viral RNA was detected by RT-qPCR.

## Results

### Mosquito collection

**Figure 1:**
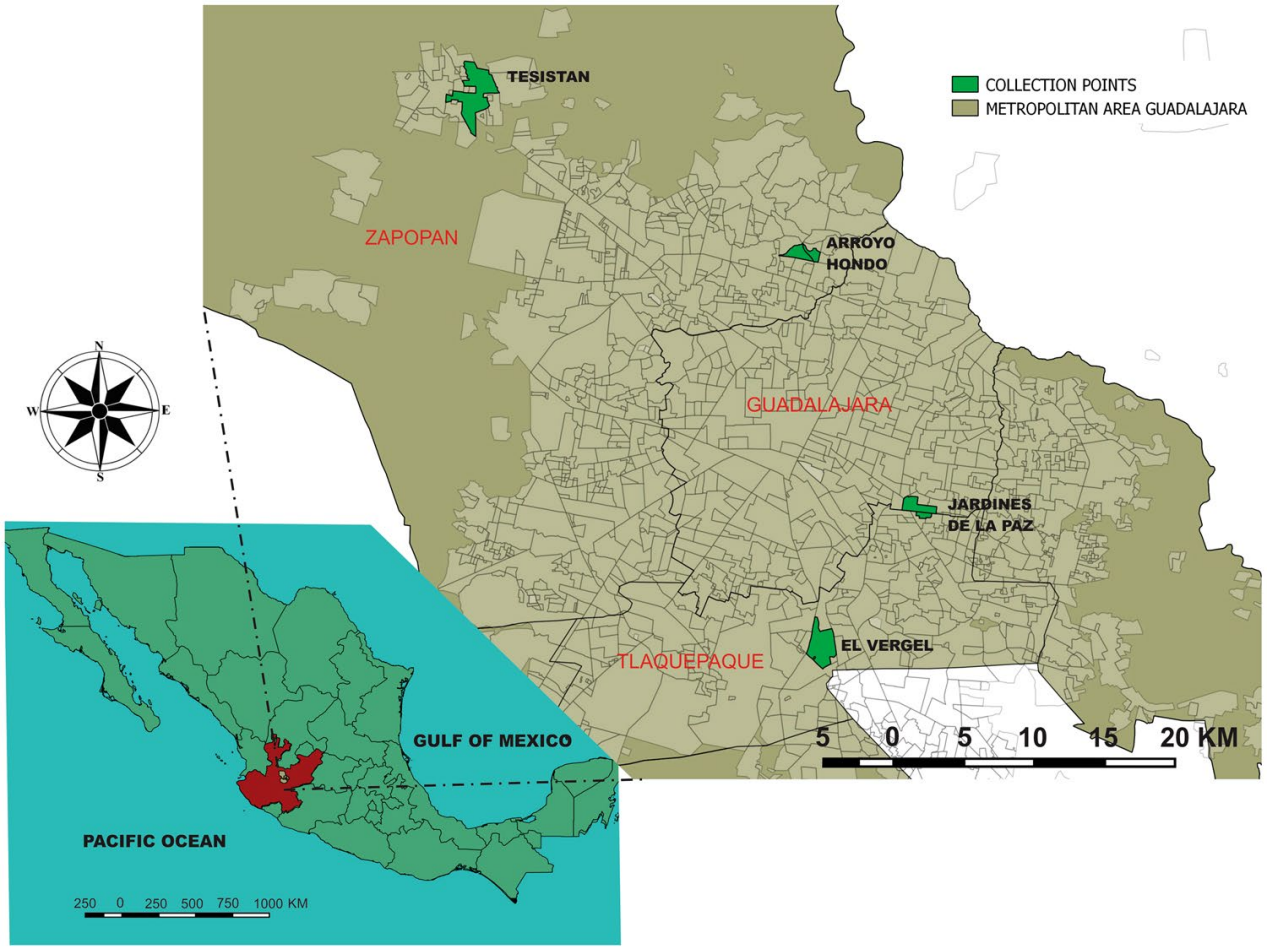
Map of the collection points in the metropolitan area of Guadalajara, Jalisco, Mexico. Maps were generated using the free and open source software QGIS Chugiak version 2.4.0 (http://www.qgis.org/es/site/about/index.html).

**Figure 2:**
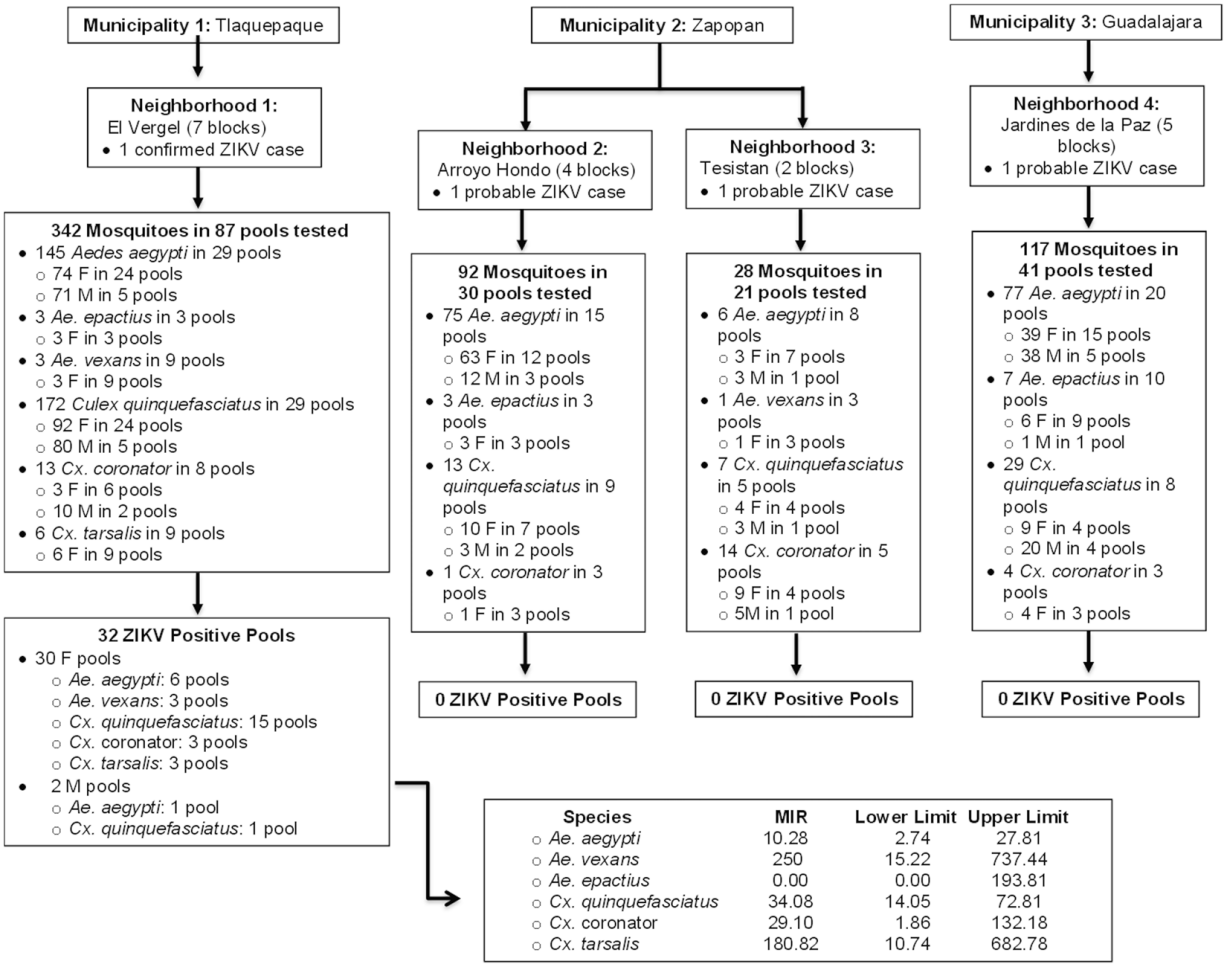
Flow chart of mosquito collection in the metropolitan area of Guadalajara, Jalisco, Mexico. The figure outlines the collection municipalities and their neighborhoods, as well as the mosquitoes collected and the pools assembled for each area. The neighborhood El Vergel was the only area with mosquitoes positive for ZIKV. Also presented is the minimum infection rate (MIR) per species and the confidence intervals. F = Female; M = Male; ZIKV = Zika Virus.

The mosquitoes were collected over five days, from September to November 2016, in 3 different municipalities (18 blocks in 4 neighborhoods) of the metropolitan area of Guadalajara (Figs 1 and 2). In this study 579 mosquitoes representing 2 genera (*Aedes* and *Culex*) and 6 species (*Ae*. *aegypti*, *Ae*. *epactius* Dyar and Knab, *Ae*. *vexans*, *Cx*. *quinquefasciatus*, *Cx*. *coronator* Dyar and Knab, and *Cx*. *tarsalis* Coquillett) were collected. The mosquitoes were then separated by the block they were collected from and by species and sex and divided into pools with a maximum of 25 insects. Female mosquitoes from these pools were dissected to separate salivary glands, midguts, and the rest of their bodies, and the dissected parts were distributed into individual tubes containing viral transport medium (Table 1).

**Table 1:** Summary of mosquito species collected in neighborhoods from the metropolitan area of Guadalajara, Jalisco, Mexico.

Number of mosquitoes of the indicated genus and species (579 total)	Female Mosquitoes			Male Mosquitoes	
	No. mosquitoes	No. assembled pools for dissection	No. pools of body parts	No. mosquitoes	No. assembled pools
*Aedes aegypti* (n = 303)	179	20	58	124	14
*Ae*. *epactius* (n = 13)	12	6	15	1	1
*Ae*. *vexans* (n = 4)	4	4	12	0	0
*Culex quinquefasciatus* (n = 221)	115	17	39	106	12
*Cx*. *coronator* (n = 32)	17	6	16	15	3
*Cx*. *tarsalis* (n = 6)	6	3	9	0	0
**Total**	**333**	**56**	**149**	**246**	**30**

### Virus isolation

**Figure 3:**
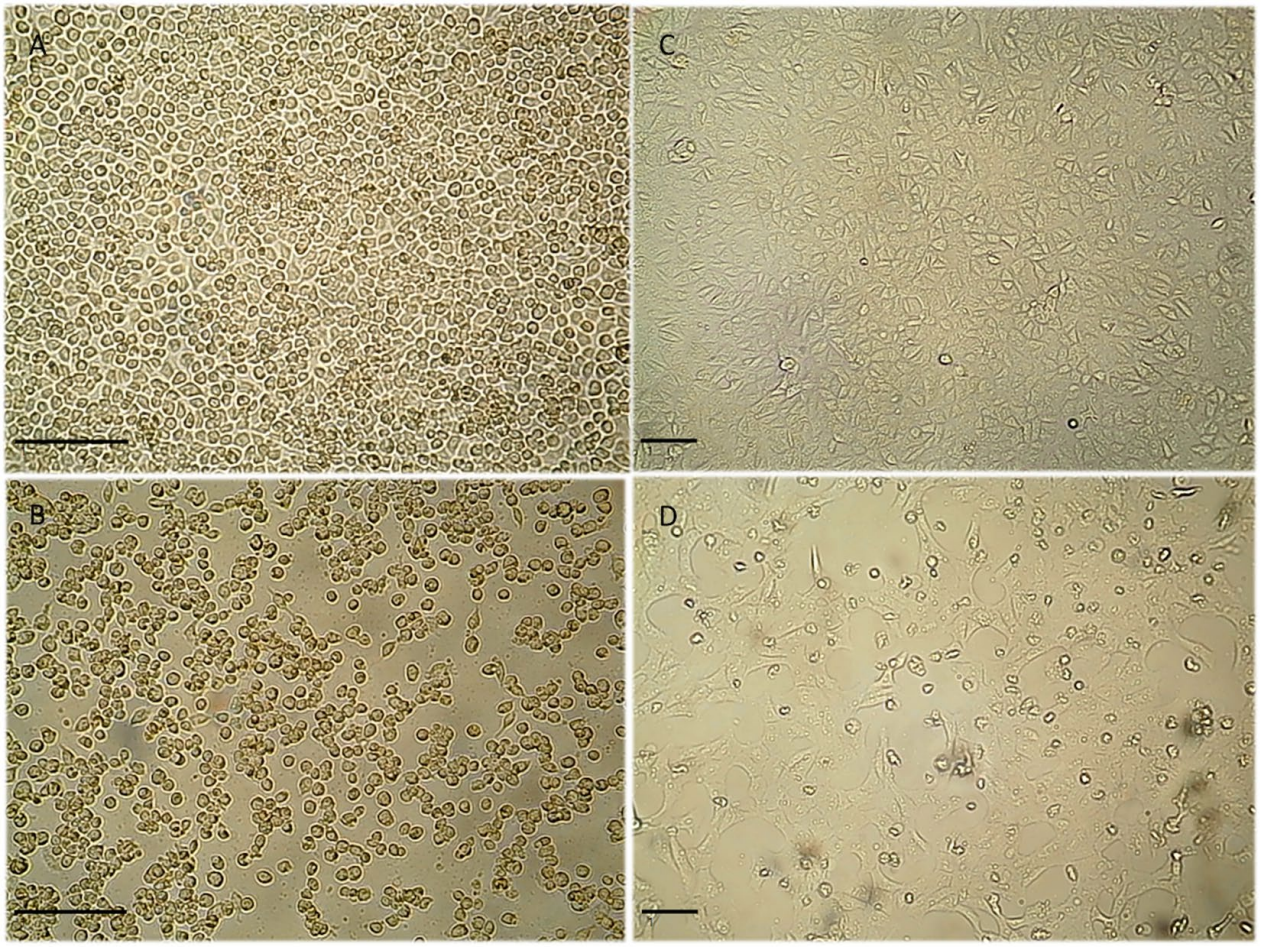
Cytopathic effect in infected and uninfected cell cultures. Cytopathic effects consisting of cell rounding, detachment and culture degeneration were observed in C6/36 and Vero cell lines at day 4 post-ZIKV inoculation, under an inverted microscope (original magnification ×20 in C6/36 and ×10 in Vero cells. Scale bars, 100 μm). (**A**) Uninfected C6/36 cells, (**B**) C6/36 cells inoculated with salivary glands homogenates showing CPE, (**C**) Uninfected Vero cells, (**D**) Vero cells inoculated with salivary glands homogenates showing CPE.

The separation and dissection of the 579 mosquitoes rendered 149 pools of female mosquito body parts and 30 pools of whole male mosquitoes (Table 1; Fig. 2), which were processed for virus isolation. A cytopathic effect (CPE) was observed in 30 of the 149 pools of female mosquito body parts; the observed CPE consisted in cell rounding, detachment, and culture degeneration in the first 1 to 5 days post inoculation (dpi) into C6/36 cell monolayers (Table 2, Fig. 3). These pools were from *Ae*. *aegypti* (2 pools), *Ae*. *vexans* (1 pool), *Cx*. *quinquefasciatus* (5 pools), *Cx*. *coronator* (1 pool), and *Cx*. *tarsalis* (1 pool). Two pools of male mosquitoes (*Ae*. *aegypti* and *Cx*. *quinquefasciatus*) also showed CPE. All the samples showing CPE represented 5 of the 6 species of mosquitoes collected.

**Table 2:** ZIKV-positive pools for the different mosquito species. CPE appearance times and Ct values by RT-qPCR.

Mosquitoes genera and species	Number of mosquitoes	Pools presenting CPE	CPE (dpi)	Ct mean values in RT-qPCR for ZIKV (SD)
*Aedes aegypti*	25	Male	2	10.50 (0.06)
*Ae*. *aegypti*	8	SG	3	14.14 (1.40)
		MG	3	13.87 (0.67)
		B	3	13.39 (0.35)
*Ae*. *aegypti*	3	SG	3	12.97 (1.07)
		MG	3	14.19 (0.95)
		B	3	14.26 (0.47)
*Ae*. *vexans*	1	SG	3	11.56 (0.91)
		MG	3	12.17 (0.56)
		B	3	13.74 (0.09)
*Culex quinquefasciatus*	25	Male	2	11.91 (1.18)
*Cx*. *quinquefasciatus*	16	SG	3	14.36 (0.33)
		MG	3	14.32 (0.35)
		B	3	14.01 (0.18)
*Cx*. *quinquefasciatus*	20	SG	3	12.76 (0.43)
		MG	5	12.16 (0.51)
		B	3	14.49 (0.14)
*Cx*. *quinquefasciatus*	25	SG	3	13.23 (0.55)
		MG	3	12.76 (0.22)
		B	3	12.59 (0.24)
*Cx*. *quinquefasciatus*	9	SG	1	12.91 (1.79)
		MG	1	12.74 (0.22)
		B	2	13.74 (0.79)
*Cx*. *quinquefasciatus*	3	SG	1	10.54 (0.71)
		MG	3	11.80 (1.19)
		B	2	14.74 (0.18)
*Cx*. *coronator*	2	SG	3	12.65 (2.69)
		MG	3	13.26 (0.40)
		B	3	13.56 (0.09)
*Cx*. *tarsalis*	3	SG	4	12.16 (0.58)
		MG	4	11.86 (0.58)
		B	1	13.89 (0.18)

All RT-qPCR reactions were performed in triplicate. Ct mean and standard deviation were calculated. SG, salivary gland; MG, midgut; B, body; CPE, cytopathic effect; dpi, days post-inoculation; SD, standard deviation.

All pools showing CPE were from the Vergel neighborhood of Tlaquepaque (Fig. 1). To confirm that the observed CPE was the result of virus infection and not a toxic component in the original samples, the cell media of inoculated cultures were re-inoculated into fresh C6/36 cells. All of the re-inoculated samples reproduced the initially observed CPE, suggesting virus growth. To discard the possibility that the isolated viruses were only entomopathogenic, the cell media from C6/36 cells were used to infect mammalian Vero cells (Fig. 3). All these inoculations produced CPE also presenting cell rounding, detachment, and culture degeneration between 4 to 6 dpi. These findings support the idea that the isolated viruses can infect mammals and discard the possibility that they are strictly entomopathogenic.

### Virus identification

Since all the re-inoculations in Vero cells produced CPE, viral RNA was extracted from the collected supernatants of the C6/36 passage, and RT-qPCR was performed. All the supernatants were ZIKV-positive, with Ct values ranging from 10 to 15 (Table 2). Even though the isolates were positive for ZIKV, the presence of chikungunya and dengue viruses in these samples was also investigated by RT-qPCR; none of the samples resulted positive for these other viruses. These results indicate that ZIKV was isolated from the mosquito pools and no chikungunya or dengue viruses were present in our samples.

### Minimum infection rates

The minimum infection rate (MIR) was calculated per 1,000 mosquitoes; MIR values of 250 for *Ae*. *vexans*, 180.82 for *Cx*. *tarsalis*, 34.08 for *Cx*. *quinquefasciatus*, 29.10 for *Cx*. *coronator*, and 10.28 for *Ae*. *aegypti* were found. The MIR values for the different mosquito species collected in this study showed high divergences, although the confidence intervals widely overlapped among them (Fig. 2).

## Discussion

To the best of our knowledge this is the first report that shows the presence of ZIKV in the salivary glands of wild-caught female mosquitoes *Cx*. *coronator*, *Cx*. *tarsalis*, and *Ae*. *vexans*, as well as and in *Ae*. *aegypti* and *Cx*. *quinquefasciatus*, which have already been reported as potential vectors for ZIKV^5–9,12,13^. All virus isolates were from mosquitoes collected in a neighborhood of the city of Guadalajara, Jalisco, Mexico, where health authorities had recorded one confirmed human ZIKV infection. No viruses could be detected from mosquitoes collected in areas where probable cases had been reported.

Different MIRs were found among the collected mosquito species using the software PooledInfRate v.4.0^14^; the highest were for *Ae*. *vexans* (250) and *Cx*. *tarsalis* (180.82) while the lowest was for *Ae*. *aegypti* (10.28) (Fig. 2). It cannot be disregarded, however, that these differences are due to the low number of collected mosquitoes for the first two species, which makes the confidence intervals overlap. Nevertheless, previous publications have experimentally evaluated the vector competence of *Ae*. *aegypti* using mosquitoes from other regions of the Americas^6^, as well as from Senegal^7^, and it was reported that these species had the lowest infection rate among those evaluated. To confirm the low MIR found in our study for *Ae*. *aegypti*, an equal number of mosquitoes per species would have to be analyzed. However, taking into consideration that *Ae*. *aegypti* mosquitoes have an endophilic and anthropophagic behavior^15^ in contrast with the other ZIKV-positive mosquito species found in this study, it could be assumed that despite the low infection rates reported by other researchers, *Ae*. *aegypti* is more likely the primary vector of the disease in the State of Jalisco, Mexico.

Most analyzed mosquito pools caused a CPE 3 dpi, but 2 pools of salivary glands of *Cx*. *quinquefasciatus* showed CPE at 1 dpi (Table 2). These findings support previously published results that suggest the *Cx quinquefasciatus* mosquito is a potential vector for ZIKV transmission^12,13^. On the other hand, the results presented in this work are discordant with previous publications reporting *Culex* spp. as poor ZIKV vectors. For instance, North American mosquito colonies maintained for decades in the laboratory were found to be non-efficient ZIKV vectors^16^. Similarly, in mosquitoes from Río de Janeiro, Brazil, *Cx*. *quinquefasciatus* were reported not to be competent to transmit the local strain of ZIKV^17^. These observations could be explained by the genetic variability of the mosquito populations, as previously suggested^7,15^. Hence, the implementation of vector competence surveillance programs should be mandatory for different geographic areas, even in the same country.

In those cases where ZIKV was found in salivary glands, a CPE was observed at a similar dpi in 5 wild-caught mosquito species, which can be then considered as potential ZIKV vectors. Nevertheless, further studies of a possible vector competence barrier to ZIKV in all mosquito species reported in this work are needed because many factors could be involved in the transmission of the virus, as has been suggested^15^. In the case of cells inoculated with salivary glands of *Cx*. *tarsalis*, a CPE was observed at 4 dpi, suggesting that this mosquito species may not be a competent vector, although it cannot be disregarded that the salivary glands in this pool had been recently infected, since the CPE in the rest of the body was observed at 1 dpi.

For the identification of the isolated viruses, RT-qPCR was performed. All the supernatants in the C6/36-infected cells were ZIKV-positive, with Ct values ranging from 10 to 15. Of particular interest, ZIKV was detected in a male pool of *Ae*. *aegypti*, supporting previous reports in mosquitoes from Brazil and in laboratory experimental infections^18–20^. In addition, we also found a ZIKV-positive male pool of *Cx*. *quinquefasciatus*, suggesting the occurrence of vertical transmission, a further cause of concern. If male mosquitoes are infected vertically, females from the same mother are probably also infected. Therefore, the number of mosquitoes with the potential to transmit the virus would increase; it will be important to address these possibilities through further studies analyzing the presence of the virus in the saliva of vertically infected female mosquitoes. Also, the finding of 3 ZIKV-infected *Culex* species (*Cx*. *quinquefasciatus*, *Cx*. *coronator*, *and Cx*. *tarsalis*) could be a major concern and potential complication for vector control programs since all these species have different breeding sites, and they could maintain viral populations during inter-epidemic periods, such as the dry season, and hibernate during colder temperatures^21^.

Recent studies indicate that ZIKV arrived in Mexico from northeast Brazil in the second half of 2014 or early 2015^22,23^. It was introduced first to the state of Chiapas in the southeast of the country and then was dispersed to 26 (out of 32) additional states^3,24^. It would be very interesting to compare the genomic sequence of our ZIKV isolates with those determined from viruses infecting humans in different regions of Mexico.

In conclusion, we found the presence of ZIKV in the salivary glands of wild-caught female mosquitoes *Cx*. *coronator*, *Cx*. *tarsalis*, *Ae*. *vexans*, *Ae*. *aegypti*, and *Cx*. *quinquefasciatus*, suggesting that these species are potential vectors for the transmission of the disease. Also, the fact that pools of male mosquitoes were found to be positive for ZIKV, suggests vertical transmission in these species. Additional studies of female mosquitoes’ saliva from the different species reported in this work are needed to confirm the presence of ZIKV and determine if they have a vector competence barrier to the virus.

## Methods

### Mosquito collection

The collection was performed by mechanical aspiration using an InsectaZooka No. 2888 A (BioQuip Products, Rancho Dominguez, CA, USA) inside residences by Personnel of the Entomological Research Unit of the Public Health Department of the State of Jalisco and the mosquitoes were transported to this research unit. Neighborhoods and blocks were selected based on the reports of the Vector Borne and Zoonotic Diseases Department of Jalisco on ZIKV confirmed or probable human cases in the area.

### Pools separation criteria

Collected mosquitoes from within each block were separated by species and sex into pools of 25 insects maximum. Female mosquitoes from each species-specific pool were dissected under a stereomicroscope. Their body parts (salivary glands, midguts, and the rest of the bodies) were distributed into individual 1.5 ml conical tubes containing 250 μL of viral transport medium (phosphate-buffered saline, pH 7.4, containing 30% fetal bovine serum and 2% of penicillin, streptomycin, and amphotericin B). Male mosquitoes were pooled without dissection, since they are not hematophagus, into 1.5 ml conical tubes containing 250 μL of viral transport medium. All pools were frozen at −20 °C to be transported to the Centro de Investigación y Asistencia en Tecnología y Diseño del Estado de Jalisco A.C. (CIATEJ).

### Virus isolation

Mosquito pools were ground, and the resultant homogenates were centrifuged at 10,000 × *g* for 10 min. Next, 25 μL of each supernatant was placed into a single well of a 24-well plate containing *Ae*. *albopictus* cells C6/36 (ATCC^®^ CRL-1660^™^). After the inoculum was adsorbed for 1 h at 28 °C, maintenance medium was added. The cell cultures were maintained at 28 °C and examined daily for evidence of viral CPE for 5 days. If no CPE was observed, the culture was freeze-thawed once and was re-inoculated in a blind passage in a fresh plate of C6/36 cells for another 5 days. If CPE was still not observed, the cultures were discarded. To confirm virus isolation, the cell media from cultures that showed CPE were re-inoculated in fresh C6/36 cells. To evaluate if these viruses could infect mammalian cells, viral passages were also performed in Vero cells (ATCC® CCL-81™), adding in both cases 25 μL of each cell media per well of a 24-well plate. After the inoculum was adsorbed for 1 h at 28 °C or 37 °C, maintenance medium was added. Cultures were maintained at 28 °C or 37 °C, for C6/36 and Vero cells, respectively, and examined daily for evidence of viral CPE. All cells observations were under an inverted microscope (Nikon Eclipse, TS100, Japan), and images were captured with a camera Optikam WiFi – 4083, and analyzed with Optika Vision Lite software 2.1 (Optika SRL, Ponteranica, Italy).

### Virus identification

After virus re-infection in Vero cells was confirmed, the isolated viruses were detected by RT-qPCR. For this, viral RNA was extracted from the cell media of the C6/36 passage that yielded CPE using a QiAmp Viral RNA Mini Kit (Qiagen™, Hilden, Germany). RT-qPCRs were carried out in a Light Cycler 480 II PCR instrument (Roche Diagnostics, Penzberg, Germany) using Verso 1-step RT-qPCR Kit (Thermo Fisher™, MA, USA). Since ZIKV, dengue, and chikungunya viruses have been co-circulating in the same area, the presence of ZIKV was determined, first, using the primer pair and probe previously reported by Lanciotti *et al*. that can detect a minimum of 25 genomic copies of the virus^25^ (Table 3). As a positive control for the reaction, we used RNA extracted from a ZIKV strain kindly provided by A.A. Sall (Institut Pasteur, Dakar, Senegal). All RT-qPCR reaction per sample were performed by triplicate. If the cell cultures showing a CPE resulted negative to ZIKV, then RT-qPCRs for chikungunya and dengue, using QuantiFast SYBR Green RT-qPCR Kit (Qiagen™ Hilden Germany), were performed. In the case of chikungunya, we used a primer pair reported by Thavara *et al*.^26^ (Table 3). For a positive control, we used RNA extracted from an isolate obtained from a patient’s serum sample, previously collected in the Hospital Civil de Guadalajara Fray Antonio Alcalde during 2014–2016^27^. In the case of dengue virus, we used the primer pair reported by Lai *et al*.^28^ (Table 3), and as a positive control, a PCR product previously cloned in our laboratory^29^. The negative controls for all the reactions were molecular biology grade water as a no template control.

**Table 3:** Primers and probes used for the identification of Zika, chikungunya and dengue viruses.

Primer name	Sequence 5′-3′	Sensitivity, no. copies reported	References
ZIKV 1086	CCGCTGCCCAACACAAG		Lanciotti, R.S., *et al*. (2007)
ZIKV 1162c	CCACTAACGTTCTTTTGCAGACAT	25	
ZIKV 1107-FAM	AGCCTACCTTGACAAGCAGTCAGACACTCAA		
CHIK-F3	(ACGCAATTGAGCGAAGCAC)	200	Thavara, U., *et al*.^26^
CHIK-B3	(CTGAAGACATTGGCCCCAC)		
DENV-For	TTGAGTAAACYRTGCTGCCTGTAGCTC	35.30	Lai Y.L., *et al*.^28^ and García-Ruíz, D., *et al*.^29^
DENV-Rev	GAGACAGCAGGATCTCTGGTCTYTC		

### Minimum infection rate analysis

We estimated the MIR per 1,000 mosquitoes, with the bias corrected by maximum likelihood estimator (MLE), with a skewness-corrected score confidence interval, using the program PooledInfRate v.4.0^14^.

## References

[1] Zanluca C (2015). First report of autochthonous transmission of Zika virus in Brazil. Mem Inst Oswaldo Cruz.

[2] CDC. Centers for Disease Control and Prevention, all countries and territories with active Zika virus transmission., (https://www.cdc.gov/zika/geo/active-countries.html) (2017).

[3] Secretaría de Salud (Mexico). Avisos epidemiológicos Zika., (https://www.gob.mx/cms/uploads/attachment/file/258140/Cuadro_Casos_ZIKA_y_Emb_sem_37_2017.pdf) (2017).

[4] Li MI, Wong PSJ, Ng LC, Tan CH (2012). Oral susceptibility of Singapore Aedes (Stegomyia) aegypti (Linnaeus) to Zika virus. PLoS Negl Trop Dis.

[5] Costa-da-Silva AL (2017). Laboratory strains of Aedes aegypti are competent to Brazilian Zika virus. PloS one.

[6] Chouin-Carneiro T (2016). Differential susceptibilities of Aedes aegypti and Aedes albopictus from the Americas to Zika Virus. PLoS Negl Trop Dis.

[7] Diagne CT (2015). Potential of selected Senegalese Aedes spp. mosquitoes (Diptera: Culicidae) to transmit Zika virus. BMC Infect Dis.

[8] Roundy CM (2017). Variation in Aedes aegypti Mosquito competence for Zika Virus transmission. Emerg Infect Dis.

[9] Ciota AT (2017). Effects of Zika virus strain and Aedes mosquito species on vector competence. Emerg Infect Dis.

[10] Gendernalik A (2017). American Aedes vexans mosquitoes are competent vectors of Zika virus. Am J Trop Med Hyg.

[11] O’Donnell KL, Bixby MA, Morin KJ, Bradley DS, Vaughan JA (2017). Potential of a northern population of Aedes vexans (Diptera: Culicidae) to transmit Zika Virus. J Med Entomol.

[12] Guedes DR (2017). Zika virus replication in the mosquito Culex quinquefasciatus in Brazil. Emerg Microbes Infect.

[13] Guo XX (2016). Culex pipiens quinquefasciatus: a potential vector to transmit Zika virus. Emerg Microbes Infect.

[14] Biggerstaff, B. J. PooledInfRate, Version 4.0: a Microsoft® Office Excel© Add-In to compute prevalence estimates from pooled samples. Centers for Disease Control and Prevention, Fort Collins, CO, USA (2009).

[15] Tabachnick WJ (2013). Nature, nurture and evolution of intra-species variation in mosquito arbovirus transmission competence. Int J Environ Res Public Health.

[16] Weger-Lucarelli J (2016). Vector competence of American mosquitoes for three strains of Zika virus. PLoS Negl Trop Dis..

[17] Fernandes RS (2016). Culex quinquefasciatus from Rio de Janeiro is not competent to transmit the local Zika virus. PLoS Negl Trop Dis.

[18] Ferreira-de-Brito A (2016). First detection of natural infection of Aedes aegypti with Zika virus in Brazil and throughout South America. Mem Inst Oswaldo Cruz.

[19] Thangamani S, Huang J, Hart CE, Guzman H, Tesh RB (2016). Vertical transmission of Zika virus in Aedes aegypti mosquitoes. Am J Trop Med Hyg.

[20] Li CX (2017). Vector competence and transovarial transmission of two Aedes aegypti strains to Zika virus. Emerg Microbes Infect.

[21] Hay SI (2000). Etiology of interepidemic periods of mosquito-borne disease. Proceedings of the National Academy of Sciences.

[22] Faria NR (2017). Establishment and cryptic transmission of Zika virus in Brazil and the Americas. Nature.

[23] Díaz-Quiñónez JA (2016). Evidence of the presence of the Zika virus in Mexico since early 2015. Virus Genes.

[24] Guerbois M (2016). Outbreak of Zika virus infection, Chiapas State, Mexico, 2015, and first confirmed transmission by Aedes aegypti mosquitoes in the Americas. The Journal of infectious diseases.

[25] Lanciotti RS (2008). Genetic and serologic properties of Zika virus associated with an epidemic, Yap State, Micronesia. Emerg Infect Dis.

[26] Thavara U (2009). Outbreak of chikungunya fever in Thailand and virus detection in field population of vector mosquitoes, Aedes aegypti (L.) and Aedes albopictus Skuse (Diptera: Culicidae). Southeast Asian J Trop Med Public Health.

[27] Resúmenes del XV concurso de trabajos libres en cartel, congreso internacional avances en medicina. *Archivos de ciencia* **8**, 37, https://www.academia.edu/31633121/Archivos_de_Ciencia (2016).

[28] Lai YL (2007). Cost-effective real-time reverse transcriptase PCR (RT-PCR) to screen for dengue virus followed by rapid single-tube multiplex RT-PCR for serotyping of the virus. J Clin Microbiol.

[29] García-Ruíz D (2016). Detection of dengue, west Nile virus, rickettsiosis and leptospirosis by a new real-time PCR strategy. SpringerPlus.

