# Author Correction: Zika Virus in Salivary Glands of Five Different Species of Wild-Caught Mosquitoes from Mexico

**DOI:** 10.1038/s41598-018-25807-9

**Published:** 2018-05-15

**Authors:** Darwin Elizondo-Quiroga, Aarón Medina-Sánchez, Jorge M. Sánchez-González, Kristen Allison Eckert, Erendira Villalobos-Sánchez, Antonio Rigoberto Navarro-Zúñiga, Gustavo Sánchez-Tejeda, Fabián Correa-Morales, Cassandra González-Acosta, Carlos F. Arias, Susana López, Rosa María del Ángel, Victoria Pando-Robles, Armando E. Elizondo-Quiroga

**Affiliations:** 1 Unidad de Biotecnología Médica y Farmacéutica, Centro de Investigación y Asistencia en Tecnología y Diseño del Estado de Jalisco, Guadalajara, Jalisco, Mexico; 2 Secretaria de Salud Jalisco, Guadalajara, Jalisco, Mexico; 3 Independent consultant in Ajijic, Jalisco, Mexico; 4 Dirección del Programa de Enfermedades Transmitidas por Vector, Centro Nacional de Programas Preventivos y Control de Enfermedades, Secretaría de Salud de México, Mexico City, Mexico; 5 Departamento de Genética del Desarrollo y Fisiología Molecular, Instituto de Biotecnología, Universidad Nacional Autónoma de México, Cuernavaca, Morelos, Mexico; 6 Departamento de Infectómica y Patogénesis Molecular, Centro de Investigación y de Estudios Avanzados del IPN, México City, Mexico; 7 Instituto Nacional de Salud Pública, Centro de Investigación Sobre Enfermedades Infecciosas (CISEI), Cuernavaca, Morelos, Mexico

Correction to: *Scientific Reports* 10.1038/s41598-017-18682-3, published online 16 January 2018

The Acknowledgements section in this Article is incomplete.

“This project was partially supported by the National Council for Science and Technology (CONACyT)-Mexico through the Mexican Network for Virology. We are indebted to Drs. José Narro Robles, Pablo Kuri Morales, and Cuitláhuac Ruiz Matus from the Secretaría de Salud of México for their help and support to carry out this work. We also thank the personnel of the Unidad de Investigación Entomológica de Occidente for their assistance in the collection of mosquitoes and separation of species. We thank Rito Gerónimo Aguirre Cortés, for the elaboration of the collection maps. We also would like to thank Brian Walsh from CIATEJ/Peace Corps for his valuable remarks in editing the English of the manuscript.”

should read:

“This project was partially supported by the National Council for Science and Technology (CONACyT)-Mexico through the Mexican Network for Virology. We are indebted to Drs. José Narro Robles, Pablo Kuri Morales, Cuitláhuac Ruiz Matus, and Jesús Felipe González Roldán from the Secretaría de Salud of México for their help and support to carry out this work. We also thank the personnel of the Unidad de Investigación Entomológica de Occidente for their assistance in the collection of mosquitoes and separation of species. We thank Rito Gerónimo Aguirre Cortés, for the elaboration of the collection maps. We also would like to thank Brian Walsh from CIATEJ/Peace Corps for his valuable remarks in editing the English of the manuscript.”

